# Serum Estradiol Levels Predict Survival and Acute Kidney Injury in Patients with Septic Shock- A Prospective Study

**DOI:** 10.1371/journal.pone.0097967

**Published:** 2014-06-06

**Authors:** Jia-Yih Feng, Kuan-Ting Liu, Edward Abraham, Cheng-Yu Chen, Po-Yi Tsai, Yu-Chun Chen, Yu-Chin Lee, Kuang-Yao Yang

**Affiliations:** 1 Department of Chest Medicine, Taipei Veterans General Hospital, Shipai Road, Taipei, Taiwan, R.O.C; 2 Institute of Clinical Medicine, School of Medicine, National Yang-Ming University, Linong Street, Taipei, Taiwan, R.O.C; 3 Taipei City Hospital, Zhongxiao Branch, Tung Teh Road, Taipei, Taiwan, R.O.C; 4 School of Medicine, National Yang-Ming University, Taipei, Taiwan, R.O.C; 5 Wake Forest School of Medicine, Medical Center Boulevard, Winston-Salem, North Carolina, United States of America; 6 Section of Chest Medicine, Department of Internal Medicine, National Yang-Ming University Hospital, Ilan, Taiwan, ROC; 7 Cardinal Tien College of Healthcare and Management, Taipei, Taiwan, ROC; 8 Department of Physical Medicine and Rehabilitation, Taipei Veterans General Hospital, Taipei, Taiwan, ROC; 9 Department of Medical Research and Education, National Yang-Ming University Hospital, I-Lan, Taiwan, R.O.C; 10 Department of Chest Medicine, Immunology Center, Taipei Veterans General Hospital, Shipai Road, Taipei, Taiwan, R.O.C; 11 Immunity and Inflammation Research Center, School of Medicine, National Yang-Ming University, Linong Street, Taipei, Taiwan, R.O.C; University of Leicester, United Kingdom

## Abstract

Sex hormones have diverse immunomodulatory effects that may be involved in the pathogenesis of sepsis. However, the roles of serum sex hormones in predicting outcomes and the severity of organ dysfunction, especially acute kidney injury (AKI), in septic shock patients remains controversial. We prospectively enrolled 107 clinically diagnosed pneumonia-related septic shock patients and serum sex hormone levels were measured on the day of shock onset. The aim of the present study was to investigate the predictive values of serum sex hormones levels for 28-day mortality and organs dysfunction, especially AKI. Compared with survivors, serum levels of progesterone (p<0.001) and estradiol (p<0.001) were significantly elevated in non-survivors. In multivariate Cox regression analysis, serum level of estradiol >40 pg/mL (p = 0.047) and APACHE II score ≥25 (p = <0.001) were found to be independent predictors of day 28 mortality. Inclusion of estradiol levels further enhanced the ability of APACHE II scores to predict survival in patients with high mortality risk. A serum level of estradiol >40 pg/mL was also an independent predictor of concomitant AKI (p = 0.002) and correlated well with severity of renal dysfunction using RIFLE classification. Elevated serum estradiol levels also predicted the development of new AKI within 28 days of shock onset (p = 0.013). In conclusion, serum estradiol levels appear to have value in predicting 28-day mortality in septic shock patients. Increased serum estradiol levels are associated with higher severity of concomitant AKI and predict development of new AKI.

## Introduction

Sepsis and septic shock involve dysregulated inflammatory responses caused by interaction between the host immune system and microorganisms. Despite recent progress in care, sepsis and septic shock remain associated with high morbidity and mortality [1], as well as diminished organ function or failure, including the kidneys, lungs, and bone marrow [2]. Among septic shock patients, 60–70% develop acute kidney injury (AKI), which is associated with elevated in-hospital mortality rates that approach 50% [3]–[5]. The RIFLE (Risk, Injury, Failure, Loss, and End-stage renal disease) classification has been proposed to define and classify AKI based on the degree of decrease in urine output and/or increase in the serum creatinine level [6]. In septic patients, RIFLE classifications were found to correlate well with disease severity and clinical outcomes, including mortality [4], [7].

Sex hormones have been reported to have regulatory effects on immune responses. Estradiol can induce the production of pro-inflammatory cytokines and macrophage activation [8], and testosterone was found to have suppressive effects on immune responses and increased susceptibility to infection [9]. Furthermore, epidemiologic studies showing that men are more likely to develop sepsis than women suggest that sex specific hormone levels may affect susceptibility to critical illness [10]. Gender disparities in outcomes following trauma or severe infection have been reported [11], [12]. Limited clinical studies also demonstrated the potential association between serum sex hormones levels and the occurrence and treatment outcomes of septic shock [13], [14]. Information concerning the association between sex hormones and sepsis-related multi-organs dysfunctions are also lacking.

Sepsis-related AKI is associated with increased mortality and morbidity in critically ill patients [4]. Recently, the beneficial effects of estradiol on ischemic AKI were demonstrated in several animal studies [15]–[17]. Therefore, the potential role of serum sex hormones on sepsis-induce AKI, as well as other organs dysfunctions, deserves further investigation. The primary aim of the present study was to investigate the predictive value of serum sex hormone levels when shock onset on outcomes in septic shock patients, and particularly on 28-day mortality. The association between serum sex hormone levels and concomitant organ dysfunction, including AKI, acute respiratory distress syndrome (ARDS), hematologic dysfunction, and metabolic acidosis, were also evaluated.

## Materials and Methods

### Ethics

The study protocol was approved by the Taipei Veterans General Hospital Institutional Review Board, and the study was conducted in accordance with the Declaration of Helsinki. Written informed consent was obtained from all participants or their authorized representatives before enrollment.

### Patients and Settings

This was a prospective, observational study conducted in a referral medical center in Taipei, Taiwan. From January 2008 to December 2011, patients admitted to the medical intensive care unit (ICU) and respiratory ICU were screened for the presence of septic shock associated with pneumonia. Specifically, patients with a diagnosis of pneumonia complicated by septic shock that fulfilled the Surviving Sepsis Campaign criteria for septic shock were included [18]. All patients had hypotension (arterial systolic blood pressure <90 mmHg or mean arterial pressure <65 mmHg despite adequate fluid resuscitation) that required treatment with vasopressor support at the time of enrollment and were recruited within 24 hours after shock onset. The diagnosis of pneumonia was defined by the presence of fever (≥38°C), leukocytosis (≥12,000/mm^3^) or leukopenia (<4000/mm^3^), increased sputum production, and new infiltrates on plain chest films [19], [20]. Patients who had underlying malignancy, autoimmune disorders, were taking sex hormone supplements, were less than 18 years old, were premenopausal women, had ARDS caused by non-pulmonary infection, and presented to ICUs with hypotension for more than 24 hours were excluded from enrollment. Demographic characteristics, underlying comorbidities, disease severity, the presence of concomitant organ dysfunction, and the Acute Physiology and Chronic Health Evaluation II (APACHE II) score were determined on the first day of enrollment. The presence of concomitant organ dysfunctions, including AKI, ARDS, hematologic dysfunction, and metabolic acidosis, were defined as previously described [7], [21]; the details of the definitions for organ dysfunction are provided in Table S1. The severity of multi-organ dysfunction was determined by the Sequential Organ Failure Assessment (SOFA) score [22]. The severity of AKI was evaluated according to the RIFLE classification system [7] in which patients were stratified into normal, risk, injury, and failure groups.

### Treatment and Outcome Evaluation

Broad-spectrum antibiotics were administered within 1 hour of the onset of septic shock, which is the standard of care in the ICUs where the current study was conducted. The antibiotics were adjusted based on clinical responses and drug susceptibility profiles of bacterial cultures. In addition, all patients were given a physiological dose of corticosteroid (hydrocortisone 200 mg/day, divided in four doses) for refractory hypotension [23]. Survival status at 28 days was set as the measure of treatment outcome.

The development of new AKI within 28 days of enrollment was recorded. New AKI was defined as increased serum creatinine >1.5 times baseline, or GFR decrease >25% of the baseline value, or decreased urine output <0.5 ml/kg/h for 6 hours in patients without concomitant AKI on enrollment.

### Blood Sampling and Sex Hormone Measurement

Peripheral blood samples were collected within 24 hours after shock onset. Total serum estradiol and progesterone were measured using radioimmunoassay (RIA) kits (Access estradiol reagent and Access progesterone reagent; Beckman Coulter Inc., Chaska, MN, USA). Serum testosterone was measured using a RIA kit (TEST-CT2, Cis Bio International, Gif sur Yvette, France).

### Statistical Analysis

Comparisons of demographic characteristics were carried out using chi-squared or Fisher’s exact tests for categorical variables and two-tailed independent t-test or Mann-Whitney U test for continuous variables. A multivariate Cox proportional hazards regression model with forward stepwise selection procedures was used to identify the risk factors for 28-day mortality. Binary logistic regression analysis was performed to determine independent variables associated with the development of concomitant AKI. Age, gender, comorbitities, and serum sex hormones levels were included in univariate analysis. A p value of less than 0.1 in the univariate analysis was required for a variable to be entered into the multivariate analysis model.

For survival analysis, patients were stratified into subgroups according to serum sex hormone levels. The Kaplan-Meier method was used to estimate survival time and the development of new AKI, and the log-rank test was used to compare mortality between subgroups of patients. Censored analysis was used because observation stopped after a patient was dead or was discharged from the hospital. Receiver operating characteristic (ROC) curves were constructed to determine the predictive abilities of sex hormone levels for survival and the presence of AKI. A p value of less than 0.05 was considered statistically significant for all tests. Statistical analysis was performed using a statistical software package (SPSS version 17.0; SPSS Inc., Chicago, IL, USA).

## Results

### Patient Characteristics

**Figure 1:**
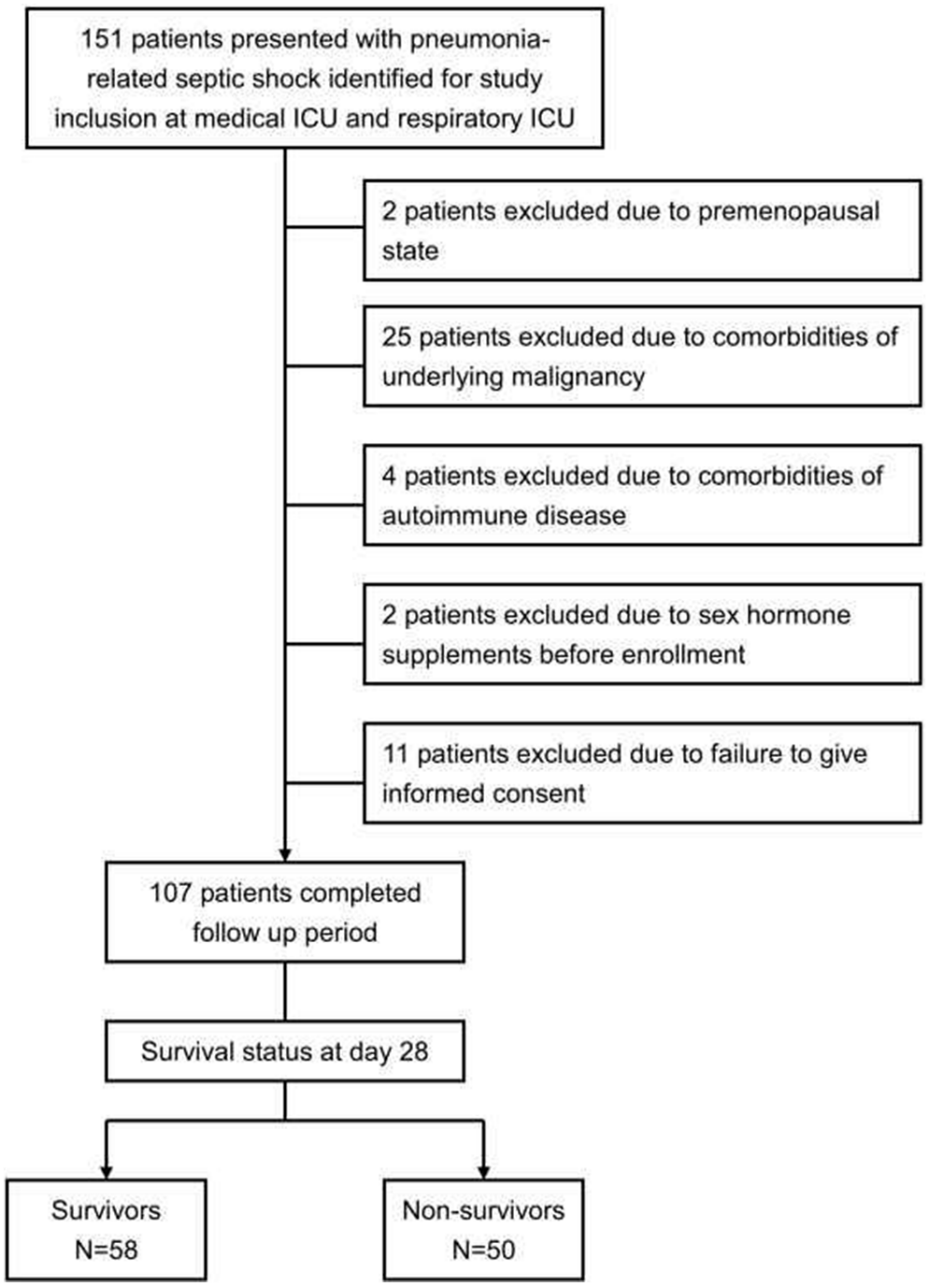
Study profile demonstrating the number of cases and reasons for exclusion.

From January 2008 to December 2011, a total of 151 pneumonia-related septic shock patients were eligible for inclusion. The study profiles and reasons for exclusion are shown in Figure 1. Finally, 107 patients were included for analysis. Among these patients, 50 died within 28 days (28-day mortality 46.7%). The demographic characteristics of the patients are shown in Table 1. The majority of the patients were male (92/107, 86%) and the mean age of the patients included in this study was 79.1±11.1 years. The mean APACHE II score was 27.8±7.5 and more than half of the patients had AKI (58/107, 54.2%) on enrollment. There were no differences in age, gender, or underlying comorbidities between survivors and non-survivors at day 28. When compared with survivors, non-survivors of pneumonia-related septic shock had higher APACHE II score (p<0.001), higher SOFA score (p<0.001), lower PaO2/FiO2 ratio (p<0.001), and a greater number of organ dysfunctions.

**Table 1:** Demographic characteristics of patients with pneumonia-related septic shock^a^.

	Overall	28-day mortality^b^		
		Survivors	Non-survivors	*p* value^c^
Patient numbers	107	57	50	
Age, years	79.1 (11.1)	78.2 (10.4)	80 (11.8)	0.41
Male gender	92 (86%)	48 (84.2%)	44 (88%)	0.57
Comorbidity				
Obstructive airway disease	25 (23.4%)	13 (22.8%)	12 (24%)	0.88
Interstitial lung disease	11 (10.3%)	6 (10.5%)	5 (10%)	0.93
Congestive heart failure	13 (12.1%)	8 (14%)	5 (10%)	0.52
Diabetes mellitus	29 (27.1%)	14 (24.6%)	15 (30%)	0.53
Chronic renal insufficiency	18 (16.8%)	8 (14%)	10 (20%)	0.41
Pathogens in sputum culture				
Gram-positive bacteria	26 (24.3%)	15 (26.3%)	11 (22%)	0.60
Gram-negative bacteria	85 (79.4%)	49 (86%)	36 (72%)	0.08
Disease severity				
APACHE II score	27.8 (7.5)	23.5 (4.6)	32.8 (7.1)	<0.001
PaO_2_/FiO_2_ ratio	190.7 (101.3)	222.6 (95.5)	156.2 (96.8)	0.001
Organ dysfunction^d^				
AKI	58 (54.2%)	25 (43.9%)	33 (66%)	0.022
Hematologic dysfunction	44 (41.1%)	19 (33.3%)	25 (50%)	0.08
Metabolic acidosis	29 (27.1%)	5 (8.8%)	24 (48%)	<0.001
ARDS	60 (56.1%)	23 (40%)	37 (74%)	<0.001
No. of organ dysfunctions				
≥2 organ failure (including shock)	90 (84.1%)	43 (75.4%)	47 (94%)	0.009
≥3 organ failure (including shock)	51 (47.7%)	15 (26.3%)	36 (72%)	<0.001
≥4 organ failure (including shock)	29 (27.1%)	4 (7%)	25 (50%)	<0.001
SOFA score	11.4 (2.7)	10.5 (2.3)	12.5 (2.8)	<0.001
Patients with second source of infection	19 (17.8%)	13 (22.8%)	6 (12%)	0.14

a Data are presented as n (%), except for age, APACHE II score, PaO2/FiO2 ratio, SOFA score, hospital LOS and ICU LOS, which are presented as mean (standard deviation).

b Pneumonia with septic shock patients were divided according to survival status at day 28.

c *p* value represents differences between survivors and non-survivors of pneumonia-related septic shock.

d Organ dysfunction was determined on the day of enrollment.

AKI, acute kidney injury; ARDS, adult respiratory distress syndrome; APACHE II, Acute Physiology and Chronic Health Evaluation II; CAP, community-acquired pneumonia; HAP, hospital-acquired pneumonia; SOFA, Sequential Organ Failure Assessment.

### Associations between Serum Sex Hormones and 28-day Mortality

**Figure 2:**
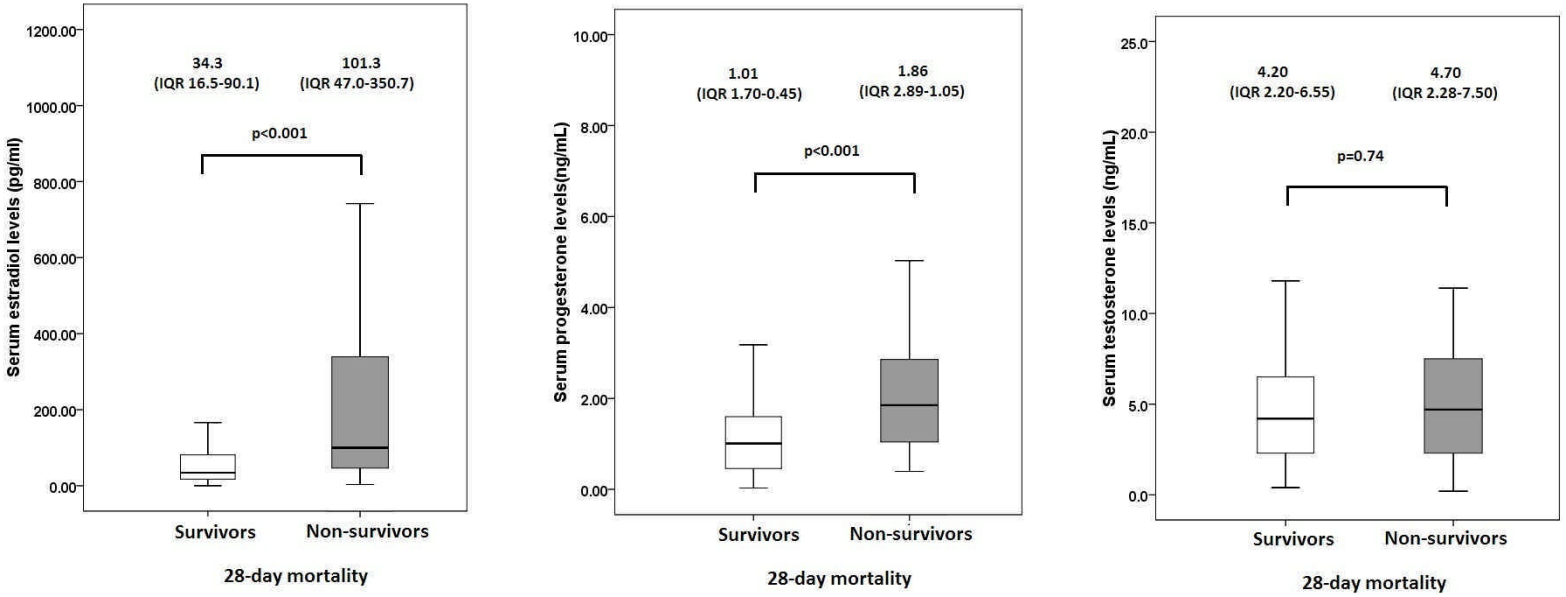
Serum sex hormone levels in patients with pneumonia-related septic shock. (A) Estradiol and (B) progesterone and (C) testosterone levels in patients with pneumonia-associated septic shock, with survivors at day 28 identified by open bars, and non-survivors at day 28 by shaded bars. Medians and interquartile ranges (IQR) are shown above each plot. Statistical significance was determined with the two-sided Mann-Whitney U test. Extreme values are not shown.

Serum sex hormone levels were comparable between male and female patients (data not shown). When compared with survivors, non-survivors of pneumonia-related septic shock patients had higher progesterone levels (p<0.001), higher estradiol levels (p<0.001), but similar testosterone levels (p = 0.74) (Figure 2).

**Figure 3:**
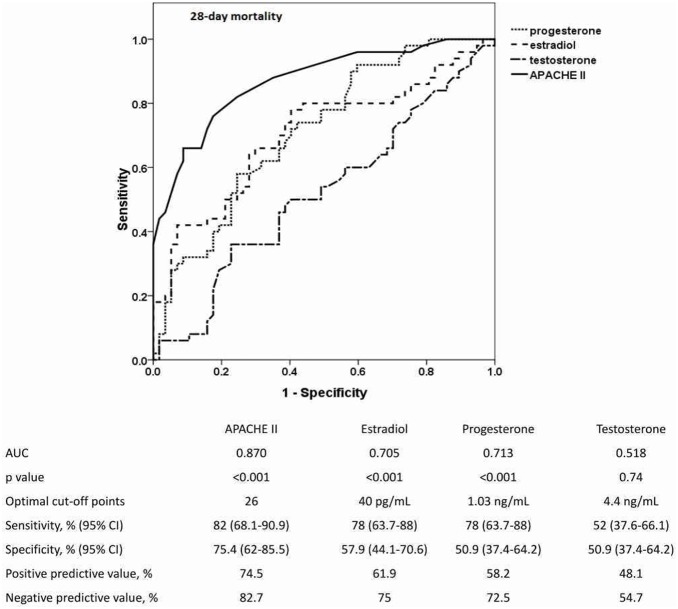
ROC curves of serum sex hormone levels and APACHE II scores for predicting 28-day mortality. The areas under the ROC curves (AUCs) for APACHE II scores, estradiol, and progesterone were significantly greater than 0.5. The optimal cutoff points for each sex hormone level and APACHE II score are listed in the attached table, along with their predictive values for the 28-day mortality of patients with pneumonia-related septic shock. ROC, Receiver operator characteristic; APACHE II, Acute Physiology and Chronic Health Evaluation II.

Regarding mortality, ROC curves of day-1 serum sex hormone levels and APACHE II scores in predicting 28-day mortality were constructed and are shown in Figure 3. The APACHE II score was a good predictor of 28-day mortality (AUC, 0.870), and the predictive value of serum levels of progesterone (AUC, 0.713) and estradiol (AUC, 0.705) were acceptable. The AUC for serum testosterone level in predicting 28-day mortality was 0.518. The optimal cutoff points of progesterone and estradiol were 1.03 ng/ml (sensitivity 78%, specificity 50.9%) and 40 pg/ml (sensitivity 78%, specificity 57.9%), respectively.

**Figure 4:**
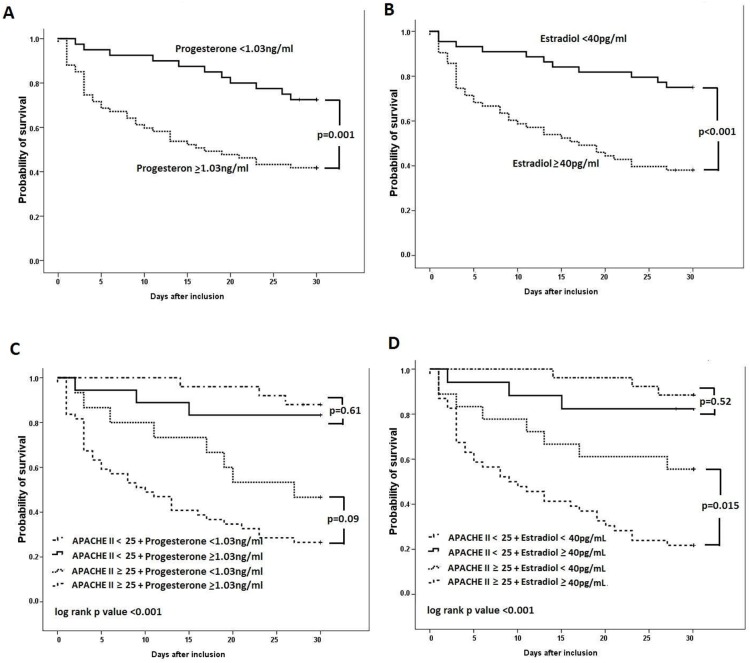
Kaplan-Meier survival curves of pneumonia-related septic shock patients, stratified by day-1 serum sex hormone levels. Patients were categorized into two groups based on the optimal cut-off points of (A) progesterone and (B) estradiol from the ROC curves. Patients were categorized into four groups based on combining (C) progesterone and (D) estradiol optimal cut-off points and high or low Acute Physiology and Chronic Health Evaluation II (APACHE II) scores. Statistical significance was tested with the log-rank test.

Kaplan-Meier survival curves categorized by the optimal cut-off points of serum sex hormone levels from the ROC curves are shown in Figure 4. Patients with higher serum levels of progesterone and estradiol had significantly higher mortality (progesterone, p = 0.001; estradiol, p<0.001). The survival curves overlapped for patients with different serum testosterone levels (p = 0.87, not shown). In order to investigate the additive value to sex hormones levels on present scoring system, we merged sex hormone levels and APACHE II scores. Kaplan-Meier analyses of survival of the subgroups of patients divided by APACHE II scores and sex hormones levels are also shown in Figure 4. In patients with an APACHE II score ≥25, combining with elevated serum estradiol levels (≥40 pg/mL) had significantly higher mortality than with lower serum estradiol levels (<40 pg/mL). (p = 0.015) (Figure 4D). In patients with an APACHE II score <25, a trend toward higher mortality was found in those with higher estradiol levels (≥40 pg/mL), but without achieving statistical significance (p = 0.52). For the survival analysis of progesterone combining with APACHE II score (Figure 4C), the survival curves separated between subgroups of patients but were all without statistical significance.

Cox regression analyses including clinical factors, serum sex hormone levels and disease severities, which were represented by APACHE II scores, associated with mortality are shown in Table 2. In multivariate analysis, serum estradiol levels ≥40 pg/mL (HR 2.04, CI 1.01–4.10, p = 0.047) and APACHE II scores ≥25 (HR 5.94, CI 2.49–14.20, p<0.001) were independent risk factors of 28-day mortality.

**Table 2:** Cox proportional hazard models for 28-day mortality prediction by comorbidities and sex hormones in patients with pneumonia-associated septic shock^a^.

	Univariate Cox Model		Multivariate Cox Model	
Variables	HR (95% CI)	*p* value	HR (95% CI)	*p* value
Age	1.01 (0.99–1.04)	0.34		
Male gender	1.20 (0.51–2.83)	0.67		
Comorbidity				
Diabetes mellitus	1.14 (0.62–2.08)	0.68		
Obstructive airway disease	1.03 (0.54–1.98)	0.92		
Interstitial lung disease	0.89 (0.35–2.24)	0.80		
Chronic renal insufficiency	1.19 (0.59–2.38)	0.63		
Congestive heart failure	0.71 (0.28–1.79)	0.47		
Estradiol (E2) >40 pg/mL	3.41 (1.74–6.67)	<0.001	2.04 (1.01–4.10)	0.047
Progesterone >1.13 ng/mL	2.83 (1.50–5.33)	0.001	1.65 (0.85–3.18)	0.14
Testosterone >4.4 ng/mL	1.10 (0.63–1.91)	0.74		
APACHE II ≥25	7.73 (3.28–18.22)	<0.001	5.94 (2.49–14.20)	<0.001

a Relative risk and 95% confidence interval were derived from the Cox proportional-hazards regression model.

HR, hazard ratio; CI, confidence interval.

### The Value of Serum Hormones in Predicting Organs Dysfunction and AKI

**Figure 5:**
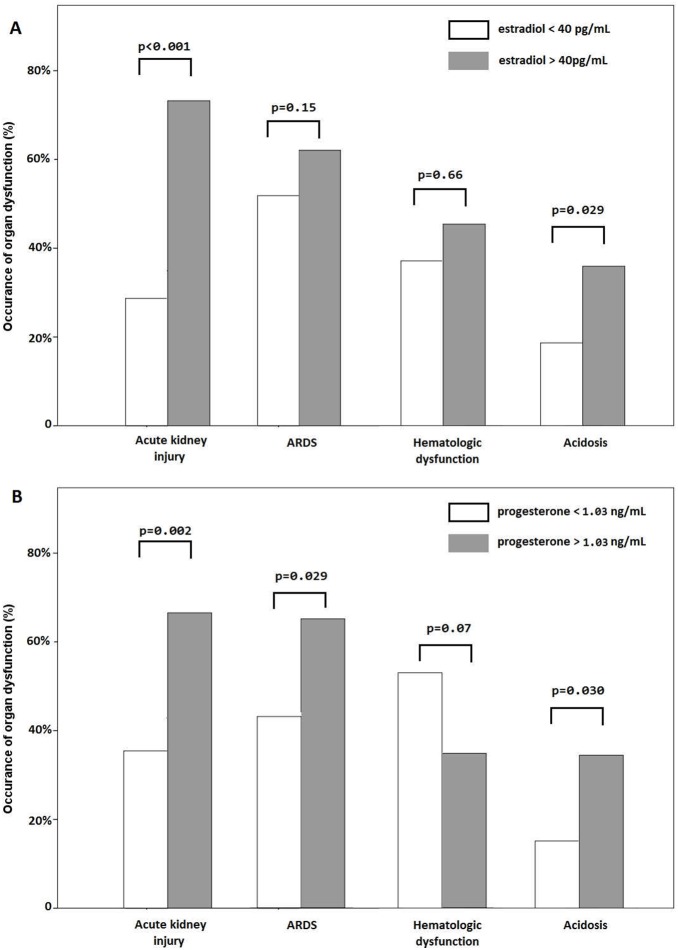
Organ dysfunctions in patients with pneumonia-related septic shock. The occurrence of organ dysfunction at the onset of septic shock was compared based on higher and lower serum (A) estradiol and (B) progesterone levels. Statistical significance was examined using the Pearson’s chi-square test. ARDS, acute respiratory distress syndrome.

Associations between the presence of organ dysfunction, including AKI, ARDS, hematologic dysfunction and metabolic acidosis, and serum sex hormone levels are shown in Figure 5. Patients with higher estradiol levels showed increased incidence of AKI (p<0.001) and metabolic acidosis (p = 0.029). Patients with higher progesterone levels showed higher incidence of AKI (P = 0.002), ARDS (p = 0.029), and metabolic acidosis (p = 0.030).

**Figure 6:**
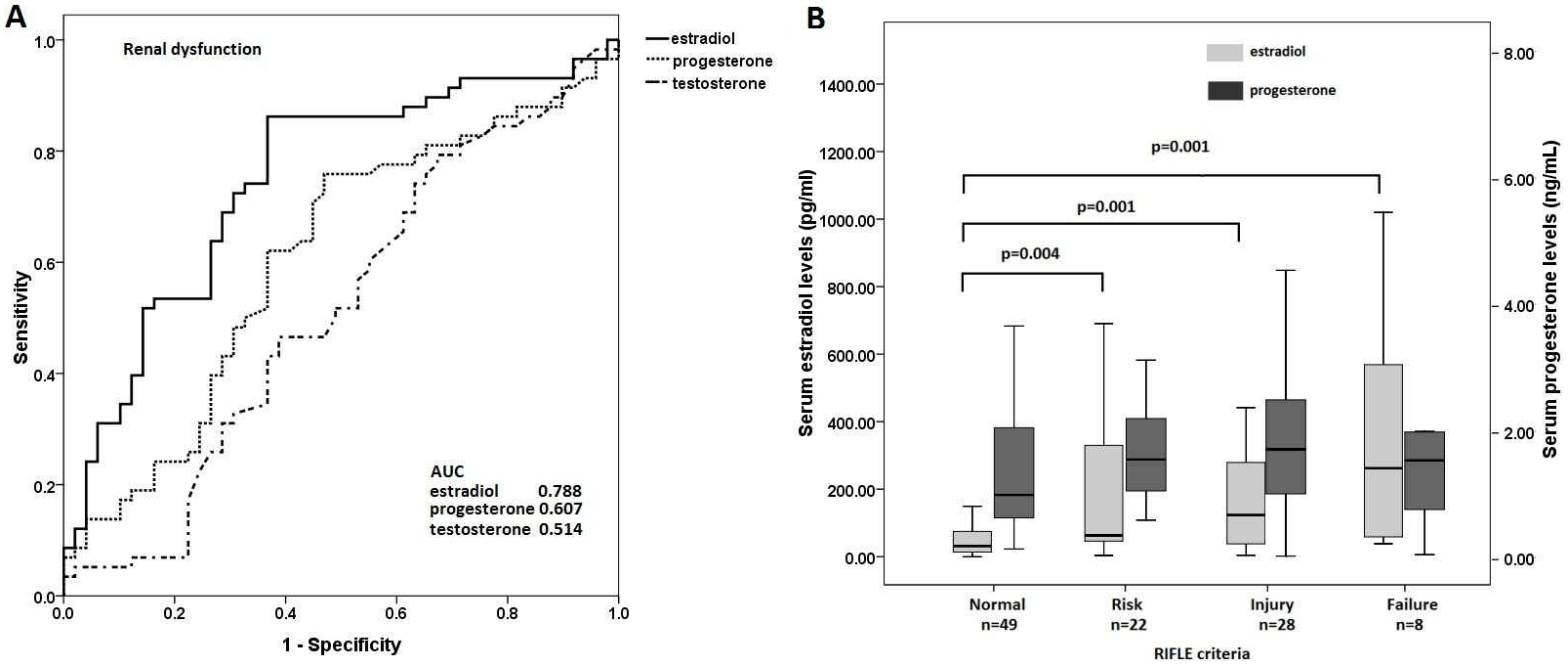
Association between serum sex hormone levels and the presence of acute kidney injury. The serum sex hormone levels are determined at the time of study enrollment, i.e. within 24 hours of the development of septic shock. (A) Receiver operator characteristic (ROC) curves of serum sex hormone levels and Acute Physiology and Chronic Health Evaluation II (APACHE II) scores for predicting AKI on enrollment. The areas under the ROC curves (AUCs) for progesterone, estradiol, and APACHE II scores were all greater than 0.5. (B) Serum estradiol and progesterone levels in pneumonia-related septic shock patients with severity of acute kidney injury stratified by RIFLE classification. Medians and interquartile ranges (IQR) of the estradiol (shaded bars) and progesterone levels (solid bars) are shown. Statistical significance was evaluated with the two-sided Mann-Whitney U test. Extreme values are not shown.

Because of the observed close correlation between serum sex hormones and AKI, we further analyzed their predictive values for concomitant AKI. ROC curves of serum sex hormones in predicting the presence of concomitant AKI are shown in Figure 6A. Serum level of estradiol was a good predictor of AKI, with AUCs of 0.788. As shown in Table 3, serum estradiol level was found to be the only independent predictor of the presence of concomitant AKI in the multivariate analysis (OR 4.73, CI 1.80–12.44, p = 0.002). APACHE II scores were not included in our multivariate analysis of concomitant AKI because serum concentration of creatinine was already in the scoring system. The mean serum estradiol and progesterone levels of patients with various levels of AKI severity on the first day of enrollment (as stratified by the RIFLE classification system) are shown in Figure 6B. Patients with more severe AKI had significantly higher estradiol levels. In contrast, there was no correlation between serum progesterone levels and severity of AKI.

**Table 3:** Univariate and multivariate analysis of predictive factors for the presence of concomitant acute kidney injury^a^.

	Univariate Analysis		Multivariate Analysis	
Variables	OR (95% CI)	*p* value	OR (95% CI)	*p* value
Age	1.05 (1.003–1.09)	0.034	1.03 (0.98–1.08)	0.26
Male gender	2.72 (0.86–8.59)	0.09	2.72 (0.74–10.03)	0.13
Comorbidity				
Diabetes mellitus	1.28 (0.54–3.03)	0.58		
Obstructive airway disease	0.89 (0.36–2.19)	0.80		
Interstitial lung disease	0.28 (0.07–1.12)	0.07	0.26 (0.06–1.21)	0.09
Chronic renal insufficiency	1.40 (0.50–3.95)	0.52		
Congestive heart failure	1.41 (0.43–4.62)	0.57		
Estradiol (E2) >40 pg/mL	5.96 (2.56–13.91)	<0.001	4.73 (1.80–12.44)	0.002
Progesterone >1.13 ng/mL	2.73 (1.24–6.02)	0.013	1.33 (0.50–3.50)	0.57
Testosterone >4.4 ng/mL	1.04 (0.49–2.23)	0.92		

a Pneumonia-related septic shock patients enrolled for analysis.

OR, odds ratio; CI, confidence interval.

**Figure 7:**
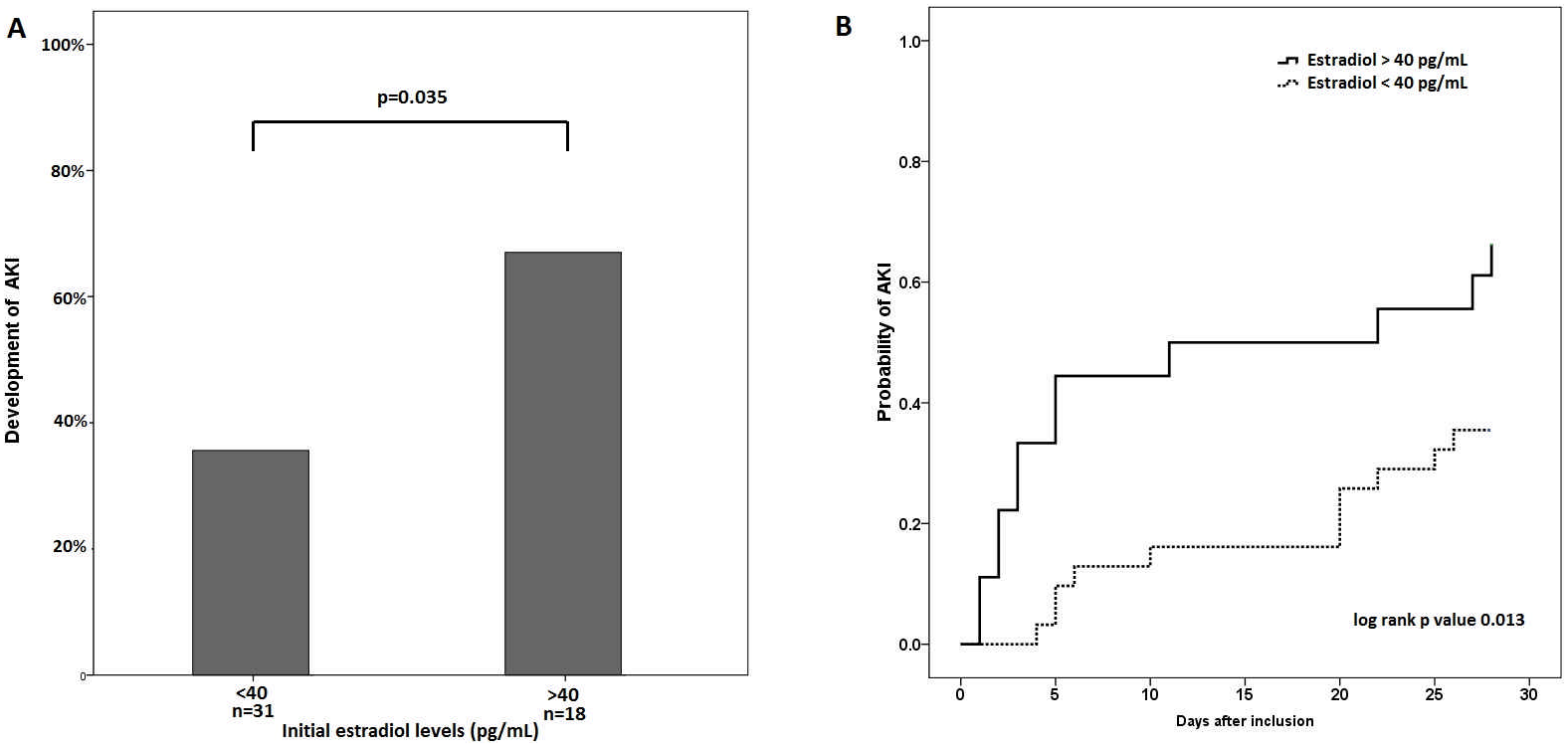
New onset of acute kidney injury within 28 days after septic shock onset. The development of new AKI between patients with higher and lower estradiol levels was compared by (A) proportion, and (B) Kaplan-Meier analysis. Statistical significance was examined with the chi-squared test and log-rank test respectively.

As our analyses demonstrated the strong association between concomitant AKI and serum estradiol levels, but not progesterone, we further evaluated the predictive value of serum estradiol for the development of new AKI. Among 49 patients who had no concomitant AKI on enrollment, AKI subsequently developed in 23 (46.9%) within 28 days after shock onset. As shown in Figure 7A, patients with serum estradiol levels ≥40 pg/mL were more likely to develop AKI (p = 0.035). Kaplan-Meier curves constructed using serum estradiol levels also showed an increased likelihood of developing AKI within 28 days of enrollment in patients with higher initial estradiol levels (p = 0.013) (Figure 7B).

## Discussion

In the present study, we found that serum estradiol and progesterone levels significantly increased in non-survivors of pneumonia-related septic shock patients. In multivariate cox regression model, only serum estradiol level was independent predictors for 28-day mortality. We also demonstrated the additive value of estradiol in combination with APACHE II scores for 28-day mortality prediction. Meanwhile, escalated serum estradiol levels correlated well with increased severity of sepsis-related AKI. In multivariate analysis, increased serum estradiol level was an independent factor associated with the presence of AKI on the first day of shock onset. Serum estradiol levels also predicted the development of new AKI within 28 days of shock onset. By contrast, there is no significant difference in testosterone levels regarding survival status and the presence of AKI.

The diverse effects of sex hormones on immune reaction and inflammation have been described extensively in cellular and animal studies. Estrogen appears to exert immunomodulatory effects through increased secretion of cytokines and chemokines from inflammatory cells [24], [25]. Estrogen can also enhance the phagocytic function of macrophages [8]. Exogenous estradiol can upregulate Th17-related inflammation and increase the severity of pneumonia in mice [26]. Both pro-inflammatory and anti-inflammatory effects for progesterone have been reported in autoimmune diseases [27]. Testosterone has been reported to have suppressive effect on immune responses, causing susceptibility to many infections [9]. One study also showed that testosterone-treated macrophages demonstrated increased susceptibility to Leishmania infection [28].

There are only a few reports evaluating serum sex hormone levels in patients with septic shock. One study demonstrated that higher levels of estradiol and estrone, but not of testosterone, were found in critically ill patients with sepsis and septic shock [13]. Another study described a trend of serially increasing serum estrogen levels in non-survivors of septic shock [14]. Consistent with these previous reports, the present study demonstrated the independent predictive value of serum estradiol, but not of progesterone and testosterone, for 28-day mortality in septic shock patients. Moreover, this study had larger sample sizes than previous studies and focused on pneumonia-related septic shock patients. Furthermore, we found that combining estradiol levels with APACHE II scores allows identification of the population at highest risk for mortality among patients with septic shock.

The source of increased estradiol concentration in patients with concomitant AKI remains uncertain. Peripheral aromatase has the ability to convert androgens into estrogens, and could be stimulated by stress [29]. Cellular study demonstrated that aromatase activity could be upregulated by inflammatory cytokines in the presence of glucocorticoid [30]. Another clinical study also reported that aromatase mRNA expression and activities were increased with elevated estrogen levels in patients underwent elective surgery [31]. The main pathway of estrogens metabolism takes place in liver and gastrointestinal tissues. Estradiol will be metabolized in liver and their conjugated form and are excreted via bile, feces and urine [32]. Estrogen conjugates can also be hydrolyzed by intestinal bacteria and excreted in the bile [33]. Therefore, the close correlation between increased serum estradiol and AKI is not simply a consequence of decreased excretion from kidney. More importantly, we identified the predictive value of estradiol level on shock onset in the development of new AKI within 28 days, which was the first report to our knowledge. Therefore, the specific role of estrogen in the pathogenesis of sepsis-related AKI deserves further investigation. Estrogen receptors are presented in the kidney, including mesangial cells, endothelium and vascular smooth muscle cells [34]. Previous experimental animal model demonstrated estrogen can activate inducible nitric oxide synthase (iNOS), leading to increased nitric oxide (NO) production that may protect the kidney from ischemic injury [15]–[17], [35], [36]. However, we did find that elevated serum estradiol levels were associated with an increased likelihood of developing AKI and a greater severity of AKI in septic shock patients. In our speculation, the possible mechanisms may lie on the complicated role of NO in septic shock. NO production is increased in endotoxemia and sepsis, and its related compounds have direct cell toxicity and contribute to profound hypotension in septic shock [37]–[39]. Therefore, despite the renoprotective effect of estrogen in ischemic renal injury, the systemic overproduction of NO in septic shock remains detrimental in sepsis-related AKI. Additionally, a recent study demonstrated a marginal association between C-reactive protein (CRP) and serum estradiol detected by immunoassay, but not in serum estradiol detected by mass spectrometry, in middle-aged and old male population [40]. Although whether the elevated estradiol levels is related to the increased CRP levels remains uncertain, we measured serum estradiol levels by RIA kit because it is still the standard method in clinical practice.

Based on our findings, what’s the practical value of serum estradiol levels in the management of septic shock patients? Although inferior to APACHE II scores, we found that estradiol has the additive value in predicting mortality when combining with APACHE II scores. Moreover, we demonstrated that estradiol could be a novel marker to predict the development of new AKI in septic shock patients. For patients with high estradiol levels, clinicians should keep adequate fluid status, avoid nephrotoxic agents, close monitor renal function and consider early dialysis if the renal function starts to deteriorate. Meanwhile, the specific role of estradiol in the severity stratification of sepsis-related AKI also deserves further investigation.

This study has several limitations worth noting. Only patients with pneumonia-related septic shock were enrolled, and most of them were relatively older with higher disease severity. The homogeneity of the patients reflects the characteristics of a tertiary medical center. Most of the enrolled patients were male, so the results might not hold true for female patients. Although middle-aged individuals were included, we did not enroll premenopausal women in order to avoid the confounding effects from menstruation. Meanwhile, since no differences in serum sex hormone levels were found between male and female patients in the present study, gender disparities in the older population studied might not be an issue. Finally, blood samples for sex hormone measurements were collected only on the first day of shock onset, limiting further evaluation of the changing patterns of sex hormone levels during the course of septic shock in predicting outcomes.

In conclusion, serum estradiol levels determined within 24 hours after the onset of pneumonia-related septic shock are predictive of 28-day mortality in this patient population. Serum estradiol levels are associated with concomitant AKI and correlates well with its severity. Initial serum levels of estradiol, but not of progesterone or testosterone, also predict the development of new AKI within 28 days of shock onset. Further studies are warranted to dissect the specific role of estradiol in sepsis-related AKI and the potential of sex hormone specific therapies in the treatment of septic shock.

## Supporting Information

Table S1 **Criteria for organ dysfunction.**
(DOC)
