# Supplementary material for: Serum Estradiol Levels Predict Survival and Acute Kidney Injury in Patients with Septic Shock- A Prospective Study
Source: PLoS One. 2014 Jun 6;9(6):e97967. doi: 10.1371/journal.pone.0097967 (PMC4048195; doi:10.1371/journal.pone.0097967)
Supplement: Table S1 — Criteria for organ dysfunction. (DOC) [file pone.0097967.s001.doc]

**Table S1.** Criteria for organ dysfunction

|  | Criteria |
| --- | --- |
| Acute kidney injury | Increased serum creatinine >1.5 times, or GFR decrease > 25% the baseline values, or decreased urine output < 0.5 ml/kg/h for 6 hours |
| Hematologic dysfunction | Platelet count < 80,000/mm^3^, or a decrease of 50% or more in the 3 days preceding enrollment. |
| Metabolic acidosis | pH ≤ 7.30 or a base deficit ≥ 5.0 mmol/liter in association with a plasma lactate level > 1.5 times the upper limit of normal. |
| ARDS | Acute onset  Bilateral infiltrates on chest radiograph  Pulmonary artery wedge pressure < 18 mmHg or lack of clinical evidence of left ventricular failure.  PaO_2_/FiO_2_ < 200 mm Hg |

ARDS, acute respiratory distress syndrome.
